# Relation of Examining Boards to State, Etc

**Published:** 1896-08

**Authors:** 


### Abstracts and Selections.

---

### Relations of Medical Examining Boards to the State, to the Schools, and to Each Other.

---

Dr. William Warren Potter, of Buffalo, President of the National Confederation of State Medical Examining and Licensing Boards, chose this title as the subject of his annual address at the sixth conference of this body, held at Atlanta, May 4, 1896.

He said there were three conditions in medical educational reform on which all progressive physicians could agree, namely: First, there must be a better standard of preliminaries for entrance to the study of medicine; second that four years is little time enough for medical-collegiate training, and third, that separate examination by a State board of examiners, none of whom is a teacher in a medical college, is a prerequisite for license to practice medicine. It is understood that such examination can be accorded only to a candidate presenting a diploma from a legally-registered school.

He further stated that a high school course ought to represent a minimum of academic acquirements, and that an entrance examination should be provided by the State for those not presenting a high school diploma or its equivalent.

He did not favor a national examining board as has been proposed, but instead thought all the States should be encouraged to establish a common minimum level of requirements, below which a physician should not be permitted to practice; then a State license would possess equal value in all the States.

In regard to reciprocity of licensure, Dr. Potter thought it pertinent for those States having equal standards in all respects to agree to this exchange of inter-state courtesy by official indorsement of licenses, but that other questions were of greater moment just now than reciprocity. Until all standards were equalized, and the lowest carried up to the level of the highest, reciprocity would be manifestly unfair.

He urges that the States employ, in their medical public offices, none but licensed physicians. This, he affirmed, would tend to stimulate a pride in the State license, and strengthen the hands of the boards.

He denied that there was antagonism between the schools and the boards, as has been asserted. He said that both were working on parallel lines to accomplish the same purpose; that there could not possibly be any conflict between them, and that they were not enemies but friends.

The medical journals of standing, from one end of the country to the other, he affirmed, were rendering great aid to the cause of reform in medical education, and the times were propitious.

He concluded by urging united effort by the friends of medical education, saying that "the reproach cast upon us through a refusal to recognize our diplomas in Europe, can not be overcome until we rise in our might and wage a relentless war against ignorance, that shall not cease until an American State license is recognized as a passport to good professional standing in every civilized country in the world."

